# Assessment of the hypothalamic‐pituitary‐adrenocortical axis function using a vasopressin stimulation test in neonatal foals

**DOI:** 10.1111/jvim.16808

**Published:** 2023-07-11

**Authors:** Erin Elder, David Wong, Katheryn Johnson, Hannah Robertson, Meghan Marner, Katarzyna Dembek

**Affiliations:** ^1^ Department of Clinical Sciences, College of Veterinary Medicine North Carolina State University Raleigh North Carolina USA; ^2^ Department of Clinical Sciences, College of Veterinary Medicine Iowa State University Ames Iowa USA; ^3^ Adobe Veterinary Center Tucson Arizona USA

**Keywords:** adrenal insufficiency, equine, equine neonates, sepsis

## Abstract

**Background:**

Bacterial sepsis is the leading cause of death in foals and is associated with hypothalamic‐pituitary‐adrenocortical axis (HPAA) dysfunction. HPAA function can be evaluated by an arginine‐vasopressin (AVP) stimulation test.

**Hypotheses/Objectives:**

Administration of AVP will stimulate a dose‐dependent rise in systemic adrenocorticotropin‐releasing hormone (ACTH) and cortisol in neonatal foals. There will be no response seen in corticotropin‐releasing hormone (CRH) and baseline AVP will be within reference interval.

**Animals:**

Twelve neonatal foals, <72 hours old.

**Methods:**

HPAA function was assessed in foals utilizing 3 doses of AVP (2.5, 5, and 7.5 IU), administered between 24 and 48 hours of age in this randomized cross‐over study. Cortisol, ACTH, CRH and AVP were measured at 0 (baseline), 15, 30, 60 and 90 minutes after AVP administration with immunoassays. The fold increase in cortisol and ACTH was calculated at 15 and 30 minutes compared to baseline.

**Results:**

All doses of AVP resulted in a significant increase in cortisol concentration over time, and a dose‐dependent increase in ACTH concentration over time. ACTH and cortisol were significantly increased at 15 and 30 minutes, respectively after all 3 doses of AVP compared to baseline (*P* < .01). There was no change in endogenous CRH after stimulation with AVP.

**Conclusion and Clinical Importance:**

Administration of AVP is safe and results in a significant rise in ACTH and cortisol in neonatal foals. A stimulation test with AVP (5 IU) can be considered for HPAA assessment in septic foals.

**Abbreviations**
AVP: arginine vasopressin
CRH: corticotropin‐releasing hormone
CIRCI: critical illness‐related corticosteroid insufficiency
HPAA: hypothalamic‐pituitary‐adrenocortical axis
MODS: multiple organ dysfunction syndrome
RAI: relative adrenal insufficiency
RIA: radioimmunoassay

## 1 INTRODUCTION

Bacterial sepsis is a major leading cause of morbidity and mortality in neonatal foals.^1^, ^2^, ^3^, ^4^ The body's response to sepsis is regulated through the hypothalamic‐pituitary‐adrenal axis (HPAA), a crucial endocrine system that attempts to maintain homeostasis.^1^, ^3^, ^4^, ^5^, ^6^ After input from the sympathetic nervous system, arginine‐vasopressin (AVP) and corticotropin‐releasing hormone (CRH) are released from the hypothalamus and elicit an increase in ACTH from the anterior pituitary.^7^ ACTH then acts on the adrenal cortex for cortisol synthesize and release.^8^, ^9^ The HPAA can become transiently impaired during times of critical illness known as relative adrenal insufficiency (RAI) or critical illness related corticosteroid insufficiency (CIRCI).^4^, ^5^, ^6^, ^10^ RAI results in abnormal cortisol production, cellular activity, and metabolism. These processes can lead to hypoglycemia, electrolyte abnormalities and hypotension.^4^, ^5^, ^10^

Prevalence of RAI in septic foals is (40%‐60%), with affected foals being more likely to develop multiple organ dysfunction syndrome (MODS) and less likely to survive.^2^, ^3^, ^11^ The gold standard diagnostic method in horses utilizes a dynamic ACTH stimulation and cortisol response test.^12^, ^13^ However, this diagnostic test has remained controversial, and produces inconsistent results in horses.^3^, ^6^, ^10^, ^11^, ^14^ This gap in our current understanding of the HPAA in horses is highlighted by the lack of a more appropriate test to evaluate HPAA function effectively.

AVP has been implicated as a contributing factor driving fluctuations in both ACTH and cortisol in horses and may be more important for short‐term control compared to CRH.^8^, ^15^, ^16^, ^17^, ^18^ We propose that AVP is an important stimulus of ACTH and cortisol production and release in neonatal foals and might be more effective in assessing HPAA function. AVP stimulation tests have been performed for HPAA assessment in cattle and demonstrated heightened peaks in ACTH and cortisol in hyperexcitable individuals.^19^ Desmopressin (DDAVP), a synthetic vasopressin analogue, is used to evaluate ACTH‐dependent Cushing's syndrome in humans, and diabetes insipidus in humans, dogs, and horses.^20^, ^21^, ^22^, ^23^, ^24^, ^25^ No studies have been conducted investigating an AVP stimulation test and HPAA response in foals.

The purpose of this study was to investigate the HPAA response to AVP in neonatal foals during the first 72 hours of life in this cross‐over study and determine a safe and effective dose of AVP for HPAA assessment. Our hypotheses were that (1) administration of AVP would elicit a dose‐dependent rise in both serum cortisol and plasma ACTH in neonatal foals, and that (2) there would be no clinically apparent adverse effects on physical examination parameters. Lastly (3), we predicted that there would be no effect on endogenous CRH and baseline AVP would be within reference interval.

## 2 MATERIALS AND METHODS

### 2.1 Animals

Twelve neonatal foals (6 healthy and 6 admitted to the hospital), 24‐48 hours of age were included in the study, with a median gestation length of 330 days (range, 322‐335 days). A statistical power analysis was performed for sample size estimation, based on data from a pilot study comparing cortisol concentration before and after administration of AVP 5 IU in healthy foals. With an alpha = 0.05 and power = 0.80, the projected sample size needed with this effect size (1.16) is approximately N = 9 per group. Thus, our sample size of N = 12 per group was adequate for the main objective of this study. Foals were recruited for the study from December 2020 to June 2021, and were determined to be not critically ill based on physical exam parameters, sepsis scores, CBC (Advia 120 Hematology Analyzer, Siemens Healthineers, Malver, Pennsylvania), and serum biochemistry analysis (Cobas C 501, Roche Diagnostics Corporation, Indianapolis, Indiana) values within acceptable reference intervals.^26^ The study sample consisted of 6 hospitalized foals managed for failure of transfer of passive immunity, flexural limb deformities, and birth via Cesarean section, and the remaining 6 were housed on their farm of birth. All foals were determined to have an intact HPAA, and no signs of systemic illness. IgG concentrations were measured using an immunoturbidimetric assay (Rapid DVM Test II, Value Diagnostics, MAI Animal Health, Melksham, Wiltshire, UK) on EDTA‐blood. Three foals were from North Carolina State University teaching herd, 3 of the foals were from Iowa State University teaching herd and the remainder of the foals (n = 6) were hospitalized at North Carolina State University Equine Hospital. The hospitalized foals were managed for their respective conditions throughout the study and none of them received exogenous corticosteroids before or during the study period.

This study was approved by the Institutional Animal Care and Use Committee and the Veterinary Clinical Research Advisory Committee of North Carolina State University and Iowa State University. Owner consent was obtained before inclusion in the study.

### 2.2 Study design

A randomized cross‐over repeated measures design was used to compare systemic CRH, ACTH and cortisol response to 3 different doses of AVP (2.5, 5, and 7.5 IU) in neonatal foals. These doses were determined based on preliminary data in this study as well as published data in horses, humans, and dogs, where higher doses of AVP resulted in mild colic signs, attributable to decreased gastrointestinal perfusion.^27^, ^28^, ^29^, ^30^, ^31^, ^32^ Injectable vasopressin (Vasostrict) was supplied as a sterile aqueous injection in glass vials (20 IU/mL), (Vasostrict, Par Pharmaceutical Companies, Inc, Spring Valley, New York). Baseline sampling was performed at 24 hours of age, and foals subsequently received 3 doses of AVP intravenously (2.5, 5, and 7.5 IU) every 12 hours. The 2.5 IU dose of vasopressin was diluted in 20 mL of sterile saline, and both 5 and 7.5 IU doses were diluted in a 250 mL bag of sterile saline for administration. Additional blood samples were collected at 15, 30, 60, and 90 minutes after each AVP infusion. The median dosage of AVP administered to each foal based on body weight was 0.06 IU/kg (range, 0.04‐0.11) for the 2.5 IU dose, 12 IU/kg (range, 0.08‐0.18) for the 5 IU dose, and 0.18 IU/kg (range, 0.12‐0.27) for the 7.5 IU/kg. The order of stimulation tests was randomized within the individual foals, and baseline AVP before each stimulation test was compared.

### 2.3 Sample collection

Jugular catheters were placed in all foals. AVP was administered over a period of 5 minutes for the 2.5 IU dose, and 10 minutes for the 5 and 7.5 IU doses. Serum and plasma were collected into serum clot and chilled EDTA‐aprotinin tubes (500 k U/mL of blood), respectively, centrifuged at 2000*g* at 4°C for 15 minutes, aliquoted, and stored at −80°C until analysis.

### 2.4 Measurement of blood hormone concentrations

Radioimmunoassays (RIA) were used to measure serum concentration of cortisol and plasma concentration of CRH (Cortisol Coated Tube radioimmunoassay kit, MP Biomedicals, LLC, Solon, Ohio; Corticotropin Releasing Hormone (CRH) [Human, Rat, Mouse, Canine, Feline] RIA Kit Assay Protocol, Phoenix Pharmaceuticals, Inc, Burlingame, California). Both cortisol and CRH RIA measurements have been previously validated in horses.^33^, ^34^ Plasma AVP concentrations were measured using a multispecies enzyme immunoassay with a detection limit of 2.11 ng/mL and a working range between 4.09 and 1000 pg/mL (Arg‐Vasopressin Enzyme Immunoassay Kit (AVP) [Multispecies], Arbor Assays, Ann Arbor, Michigan). Plasma samples were extracted with the provided extraction solution following the manufacturer's instructions, before running the kit. The AVP intra‐assay and inter‐assay CVs were 8.9% and 8.6%, respectively. Plasma ACTH was measured using an automated chemiluminescent assay (CLIA), previously validated for equids (ACTH Immulite kit, Siemens Medical Solutions USA, Tarrytown, New York).^35^

### 2.5 Data analysis

Data were tested for normality by a Kolmogorov‐Smirnov test and were noted to be not normally distributed. Data were presented as median and interquartile ranges. The Freidman's test was used to compare cortisol, ACTH, and CRH concentrations at baseline and 15, 30, 60, and 90 minutes after each dose of AVP to assess for the effect of time or dose of AVP on hormone concentration. Dunn's post hoc comparisons were made when relevant. A fold increase was defined as the ratio of an increased concentration at 15 and 30 minutes to the baseline concentration. Peak ACTH and cortisol concentration was defined as the single highest hormone concentration achieved in each individual foal. Time of peak cortisol and ACTH concentration was the time point at which peak hormone concentration was achieved in each foal for each dose of AVP. Differences in peak concentration and time of peak for cortisol and ACTH were analyzed with Kruskal‐Wallis test with Dunn's post hoc test for multiple comparisons. Differences in fold increase in cortisol and ACTH concentration were analyzed with the Friedman's test with a Dunn's post hoc test for multiple comparisons. There was no effect of time or dose of AVP on endogenous CRH concentration in foals. Consequently, the fold increase as well as peak CRH and time of peak CRH were not calculated.

Spearman's rank correlation test was used to determine the effect of body weight on fold increase in cortisol and ACTH concentration 15 and 30 minutes after AVP stimulation. The cortisol and ACTH fold increase at 15 and 30 minutes after AVP stimulation were compared with the Friedman's test to determine the effect of order on dosing AVP. The cortisol and ACTH fold increase at 15 and 30 minutes were compared between foals presented to the hospital and healthy foals with 3‐way ANOVA (fold increase, dose of AVP, and group of foals) and no difference was noted between 2 groups of foals. A comparison was made between these 2 groups to further confirm them as not critically ill, and to demonstrate an appropriately functioning HPAA. Relationships between categorical variables were analyzed using contingency tables and Fisher's exact test. Statistical analysis was performed using Prism and IBM SPSS Statistical Software (SPSS and GraphPad Software, Inc [version 7.0b], La Jolla, California).

## 3 RESULTS

### 3.1 Study sample

The study sample consisted of 12 neonatal foals (5 females, 7 males), 7 Quarter Horses, 2 Fjords, 2 Thoroughbreds and 1 Arabian evaluated at 24 hours of age. Mean body weight was 41.4 kg (+ − 18.5 kg, range, 22‐60 kg). All foals completed the AVP stimulation test, and no clinically apparent adverse effects attributed to administration of AVP were identified at all 3 doses. A mild, transient drop in baseline heart rate with a median heart rate of 81.3 bpm (range, 72.3‐95), 83.0 bpm (range, 73.6‐99.23), and 84.58 bpm (range, 73.6‐106.15), was detected after administration of 2.5, 5, and 7.5 IU of AVP, respectively. Additionally, mucous membrane color was slightly paler for 10‐20 minutes after administration of AVP and normalized by time 30 minutes. Median sepsis score was 2.5 (range, 0‐7),^26^ segmented neutrophil count was 5899 cells/μL (range, 1790‐9632), blood lactate concentration was 2.4 mmol/L (range, 1.1‐5.3), blood glucose concentration was 143 mg/dL (range, 88‐240), fibrinogen concentration was 245 mg/dL (range, 200‐400), and plasma IgG concentration was 630 mg/dL (range, 87‐1776). Blood culture results were available in 7 foals, with 2/7 having positive growth from 1 or more sites. One foal had 1+ growth of *Enterococcus faecium* from the sample obtained from the jugular vein, and 1+ growth of a *Corynebacterium* spp. from the sample obtained from the cephalic vein. The second foal had growth of *Stenotrophomonas rhizophila* from jugular vein site, and no growth from an alternative location. Possible explanations for positive blood culture in conjunction with the low sepsis scores and no evidence of sepsis based on physical exam variables and blood work include sterile site contamination during blood collection, or a transient bacteremia that occurs in healthy neonatal foals.^36^

### 3.2 Endogenous AVP concentration before AVP stimulation tests

Baseline endogenous AVP concentration was 9.5 (5.8‐13.2), 10.9 (7.9‐13.4), and 8.8 (7.11‐14.16) pg/mL before stimulation with 2.5, 5, and 7.5 IU of AVP, respectively. There were no differences detected in basal AVP concentration before stimulation test with each dose of AVP (*P* > .05).

### 3.3 Serum cortisol concentrations at 0, 15, 30, 60, and 90 minutes after AVP stimulation in neonatal foals

**FIGURE 1:**
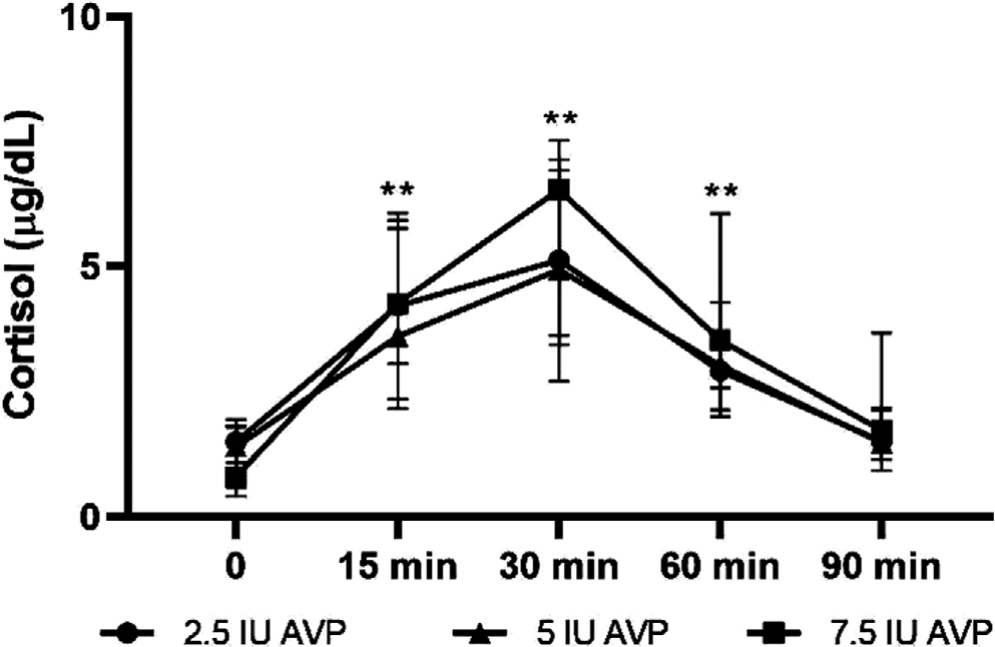
Serum cortisol concentrations before (time 0) and 15, 30, 60 and 90 minutes after intravenous administration of 2.5, 5, and 7.5 IU of AVP in neonatal foals, <72 hours old (median and interquartile ranges). **P < .01 compared to time 0.

Serum cortisol concentrations before and after AVP stimulation in neonatal foals are presented in Figure 1. Endogenous cortisol concentration was 0.93 (0.18‐3.06), 0.44 (0.21‐1.16), and 0.19 (0.17‐2.1) μg/dL before stimulation with 2.5, 5, and 7.5 IU of AVP, respectively. There was a significant effect of time on cortisol concentration after administration of 2.5, 5, and 7.5 IU of AVP in neonatal foals. Serum cortisol concentration increased 15, 30, and 60 minutes after administration of all 3 doses of AVP compared to baseline (*P* < .01). There was no statistically significant effect of AVP dose on cortisol response (*P* > .05).

### 3.4 Plasma ACTH concentrations at 0, 15, 30, 60, and 90 minutes after AVP stimulation in neonatal foals

**FIGURE 2:**
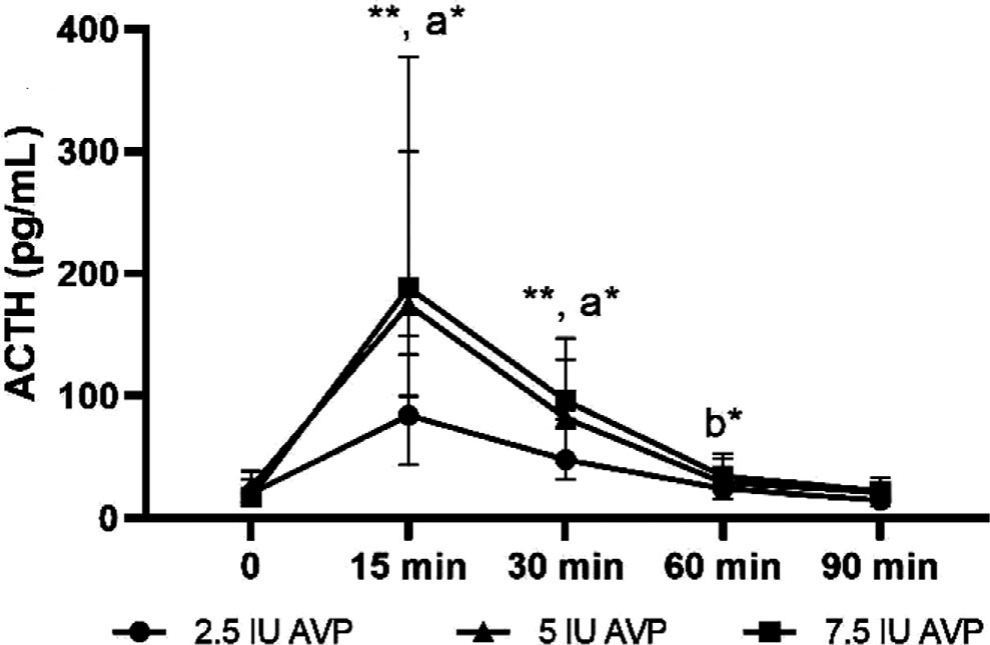
Plasma ACTH concentrations before (time 0) and 15, 30, 60 and 90 minutes after intravenous administration of 2.5, 5, and 7.5 IU of AVP in neonatal foals, <72 hours old (median and interquartile ranges). **P < .01 compared to time 0; a*P < .05 7.5 compared to 2.5 IU dose of AVP within the same time point; b*P < .05 compared to time 0 for 7.5 IU of AVP.

Plasma ACTH concentrations before and after AVP stimulation in neonatal foals are presented in Figure 2. Endogenous ACTH concentration was 20.3 (14.9‐31.48), 25.6 (15.2‐38.4), and 17.1 (15.8‐24.45) pg/mL before stimulation with 2.5, 5, and 7.5 IU of AVP, respectively. There was a significant effect of both time and dose of AVP on ACTH concentration after administration of 2.5, 5, and 7.5 IU of AVP. Plasma ACTH concentration increased 15 and 30 minutes after administration of all 3 doses of AVP compared to baseline (*P* < .01). For the 7.5 IU dose of AVP, plasma ACTH concentration was higher at 15 and 30 minute time points compared to 2.5 IU of AVP (*P* < .05). Plasma ACTH concentration remained higher at 60 minutes compared to time 0 in response to 7.5 IU of AVP (*P* < .05).

### 3.5 Change in systemic ACTH and cortisol concentrations after administration of 2.5, 5, and 7.5 IU of AVP in neonatal foals

**FIGURE 3:**
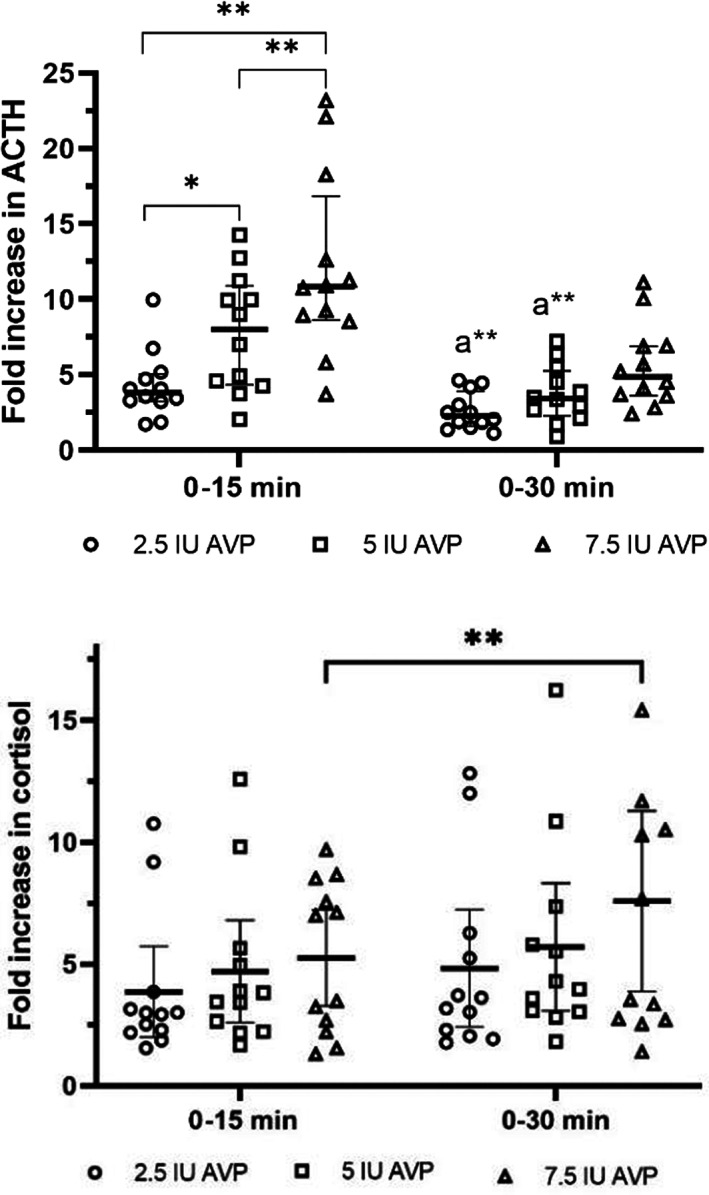
Change in cortisol (A) and ACTH (B) concentration 15 and 30 minutes after administration of 2.5, 5, and 7.5 IU of AVP (median and interquartile ranges). *P < .05; **P < .01; a—compared to 0‐15 minutes in the same dose of AVP.

Change in systemic ACTH and cortisol concentration 15 and 30 minutes after AVP stimulation is presented in Figure 3A,B, respectively. ACTH response 15 minutes after AVP stimulation with 5 and 7.5 IU of AVP was higher compared to 2.5 IU dose of AVP (*P* < .05) and (*P* < .01), respectively. For the 7.5 IU of AVP, the fold increase in ACTH concentration 0‐15 minutes was higher compared to 5 IU dose of AVP (*P* < .01). Fold increase in ACTH 0‐15 minutes in response to 5 and 7.5 IU of AVP was higher than the fold increase in ACTH 0‐30 minutes for the same doses of AVP (*P* < .01). There were no differences detected in the fold increase in cortisol concentration from baseline to 15 and 30 minutes after stimulation with all 3 doses of AVP (*P* > .05). Fold increase in cortisol concentration 0‐30 minutes after administration of 7.5 IU of AVP was higher compared to the fold increase in cortisol concentration 0‐15 minutes after the same dose of AVP (*P* < .01). The order of AVP dosing had no effect on the fold increases in cortisol and ACTH at 15 and 30 minutes after stimulation (*P* > .05). There was no statistical difference in cortisol and ACTH response to AVP among healthy foals and foals admitted to the hospital (*P* > .05). Finally, there was no effect of body weight on fold increase of cortisol and ACTH at 15 and 30 minutes after stimulation with all 3 doses of AVP (*P* > .05).

### 3.6 Peak concentration and time to peak for cortisol and ACTH in neonatal foals

Peak and time to peak for cortisol and ACTH concentrations in neonatal foals are presented in Table 1. The time to peak for cortisol concentration was higher for 7.5 IU of AVP compared to 2.5 IU (*P* < .05). However, the cortisol peak concentrations were not different between 3 doses of AVP. Peak ACTH concentration was higher for 7.5 IU of AVP compared to 2.5 IU and occurred at 15 minutes after the stimulation (*P* < .01).

**TABLE 1:** Cortisol and ACTH peak and time to peak after AVP stimulation (2.5, 5, and 7.5 IU) in 12 neonatal foals <72 hours old (median and interquartile ranges).

Cortisol		
AVP dose	Time to peak (min)	Peak cortisol (μg/dL)
2.5	30 (15‐30)	5.33 (3.6‐7.5)
5	30 (30‐30)	4.9 (2.8‐6.9)
7.5	30 (30‐52.5)	6.54 (2.13‐7.4)

ACTH		
AVP dose	Time to peak (min)	Peak ACTH (pg/mL)
2.5	15 (15‐15)	84.15 (43.25‐133.4)
5	15 (15‐15)	174 (99.8‐485)
7.5	15 (15‐15)	188.5 (149.5‐433)^a^ ^,^ **

^a^ Compared to 2.5 IU.

** *P* < 0.01.

### 3.7 Plasma CRH concentrations at 0, 15, 30, 60, and 90 minutes after AVP stimulation in neonatal foals

Endogenous CRH concentration was 12.3 (7.04‐24.6), 10.07 (7.9‐24.9), and 10.3 (7.08‐23.4) pg/mL before stimulation with 2.5, 5, and 7.5 IU of AVP, respectively. Following administration of all 3 doses of AVP, no change in CRH was produced.

## 4 DISCUSSION

This study investigated AVP stimulation tests in foals using a safe and effective dose for assessment of the HPAA. The results show that all 3 doses of AVP produced a significant rise in both ACTH and cortisol at 15 and 30 minutes, respectively, demonstrating AVP is a potent regulator of both hormones in foals.^8^, ^16^, ^37^, ^38^ This is contrary to AVP stimulation in humans, where no significant change is seen in either hormone.^39^ Peak in ACTH was more pronounced for the 7.5 IU dose compared to the 2.5 IU dose, suggesting a dose‐dependent effect between AVP and ACTH. After the increase in ACTH, peak cortisol response was seen with all 3 doses of AVP at 30 minutes, which is expected and consistent with reports documenting the relationship between the 2 hormones.^6^, ^11^, ^40^, ^41^, ^42^ The cortisol fold increase was significant at all 3 doses; however, the highest dose produced an even more pronounced fold increase, close to a 7‐fold increase from baseline.

ACTH stimulation tests are still considered to be the gold standard for RAI or CIRCI detection in critically ill foals and people.^3^, ^6^, ^10^, ^11^ However, exogenous ACTH stimulation tests have resulted in a variable cortisol response in both healthy and septic foals, and critically ill humans, implicating it as a poor diagnostic tool in both species.^1^, ^4^, ^5^, ^11^, ^41^, ^43^ In the current study, exogenous AVP administration not only resulted in a rise in endogenous ACTH, but also produced a significant increase in cortisol. The cortisol fold increase in this study is significantly higher after exogenous AVP administration compared to the cortisol fold increase after exogenous ACTH administration in previous studies in foals.^11^, ^41^ Direct comparison between the AVP stimulation and previously investigated doses of ACTH is challenging as these are 2 different hormones with different mechanisms of action. A significant difference has been demonstrated between 1 and 10 μg of ACTH in cortisol response at 30 minutes, but no difference between 100 and 250 μg of ACTH in overall cortisol response until 120 minutes was reached.^44^ Our findings suggests that AVP is not only a potent stimulant of pituitary ACTH release, but similar to rats and humans, AVP in horses potentially acts on V1a receptors on the adrenal cortex directly stimulating cortisol synthesis and release.^7^, ^29^, ^45^, ^46^, ^47^, ^48^ This additional and direct pathway is a possible explanation for the heightened cortisol peak seen with AVP stimulation compared to ACTH stimulation. Future studies are needed to directly compare AVP and ACTH stimulation tests in a cross‐over design, in the same study sample of foals.

Baseline ACTH and cortisol concentrations are within the reference interval in this study sample and are consistent with a healthy and functionally intact HPAA.^3^, ^6^, ^34^, ^42^, ^44^ The peak in both hormones, however, was more pronounced in foals than in previous studies in adult horses.^15^ Administration of 5‐10 IU of exogenous AVP in healthy adult horses resulted in a 55.2% (range, 38.5‐71.9) rise in ACTH from baseline, which is lower compared to our findings in foals.^15^ This suggests that neonatal foals might be more sensitive to AVP stimulation than adult horses, however, more studies are needed to compare HPAA response to AVP in adult horses and foals. Septic foals and those with a dysfunctional HPAA, as defined by RAI, are likely to have a blunted and poor response of both ACTH and cortisol after AVP administration. Administration of 100 μg of exogenous ACTH in healthy foals 36‐48 hours of age, results in a cortisol fold change of 4.5 (2‐9.5) with delta cortisol (change in cortisol from baseline) at 30 minutes of 6.8 μg/dL.^11^ Administration of 10 μg of exogenous ACTH in healthy and hospitalized foals results in delta cortisol at time 30 minutes being 24% (−88 to 345.8) and 16% (−91 to 295) for the 2 groups of sick foals and 125% (1.3 to 316) for the healthy foals.^41^ Again, there is a poor cortisol response after ACTH stimulation in healthy, unstressed foals.^3^, ^11^, ^41^, ^44^ This is juxtaposed to our study that demonstrates a reliable and pronounced rise in cortisol with even the low dose (2.5 IU) of AVP. Our study shows that AVP is a potent HPAA stimulant on multiple levels, resulting in ACTH and cortisol release in foals.

Baseline cortisol, AVP, ACTH, and CRH concentrations were within the reference interval for all foals, further supporting their status as healthy.^6^, ^34^, ^42^, ^44^ After AVP administration, no response was detected in CRH, indicating that AVP has a negligible effect on the hypothalamic release of CRH in neonatal foals. Additionally, a drop in CRH was not demonstrated throughout the rise of ACTH and cortisol, which would be expected because of the negative feedback loop of both hormones.^7^ However, the majority of CRH is released into the hypothalamic‐hypophyseal portal vessels, as opposed to systemic circulation, which is a possible explanation for this finding.^6^ CRH is considered to be the main stimulus driving ACTH release in healthy humans, however, close correlation between these hormones in horses only occurs during times of critical illness such as with profound hypoglycemia.^17^ In healthy horses, AVP and ACTH release is tightly correlated with up to 92% of ACTH peaks occurring subsequent to an AVP peak even in the face of a stable pituitary venous CRH.^17^, ^18^ In addition to an unchanged CRH seen in our study, no effect on baseline AVP was detected with previous AVP administration. Stimulation tests were performed both in the morning and the evening with no effect on the time of day seen in the cortisol responses, which is expected in foals of this age. Foals inherently lack a circadian rhythm until they are 20‐40 days of age, at which point they develop a diurnal rhythm.^49^, ^50^

The objective of this study was to investigate if an AVP stimulation test is an appropriate diagnostic tool to stimulate the HPAA and identify critically ill foals with RAI. We demonstrated that AVP reliably produces a significant HPAA stimulation in neonatal foals, with resulting rise in both cortisol and ACTH, as compared to previously published studies in adult horses.^8^, ^11^, ^15^, ^42^ AVP stimulation with the 5 IU dose is ideal for assessment of both pituitary and adrenal responses, as demonstrated by this study. 5 IU of AVP produced a significantly higher ACTH peak at 15 minutes compared to the 2.5 IU dose; whereas the 7.5 IU dose did not produce any clinically significant difference in either ACTH (8 vs 10‐fold increase) or cortisol, compared to the 5 IU dose. Dynamic evaluation of both cortisol and ACTH, additionally can be used to differentiate between primary and secondary adrenal insufficiency for more targeted therapy. Future investigation into HPAA response to exogenous AVP in critically ill foals compared to healthy foals, and those that have a dysfunctional HPAA is warranted to establish cut‐off values for cortisol and ACTH response to diagnose RAI.

No clinically important adverse effects were observed with the 3 doses of AVP (2.5, 5, and 7.5 IU). A slight drop from baseline heart rate and pale mucous membranes were detected with all 3 doses; however, these changes were transient and persisted no longer than 15 minutes.^45^, ^51^ There was no significant effect of dose on duration or severity of decline in heart rate.^5^

Limitations of this study include the small sample size (n = 12). The cross‐over design minimized this limitation to improve statistical power. A second limitation was that 6 of the foals were subjected to stimulation while in hospital. However, there is much evidence from clinical examination, clinicopathologic data, sepsis scores and baseline hormone levels that support their healthy status. Statistical comparison showed there was no difference in cortisol and ACTH response between healthy foals and foals admitted to the hospital. However, the main goal of this study was to investigate HPAA response to AVP in healthy foals, and data regarding critically ill and septic hospitalized foals are warranted for comparison of hormone response levels between groups. A third limitation considered is the positive blood culture results identified in 2 foals, which may be interpreted as evidence of sepsis. Alternative explanations for this include site collection contamination or transient bacteremia that has been documented in otherwise healthy foals.^36^ Another limitation is that the 5 and 7.5 IU doses were diluted in 250 mL of saline and administered over 10 minutes, compared to a smaller dilution and more rapid infusion of the 2.5 IU dose (5 minutes). Initiation of blood collection began 15 minutes after the start of the infusion and potentially could have resulted in variation between peak hormone concentrations between the doses. Finally, foals were not monitored after the 90‐minute study period for any possible delayed adverse effects. However, rapid plasma clearance of AVP is expected because of its short half‐life of 24 minutes, and delayed adverse effects are unlikely.^51^

## CONFLICT OF INTEREST DECLARATION

Authors declare no conflict of interest.

## OFF‐LABEL ANTIMICROBIAL DECLARATION

Authors declare no off‐label use of antimicrobials.

## INSTITUTIONAL ANIMAL CARE AND USE COMMITTEE (IACUC) OR OTHER APPROVAL DECLARATION

Approved by North Carolina State University IACUC, policy number 20‐534.

## HUMAN ETHICS APPROVAL DECLARATION

Authors declare human ethics approval was not needed for this study.
